# Same hydrolase knockouts, different outcomes: CRISPR/Cas9 editing workflow shapes resulting cellular phenotype and transcriptome in CHO

**DOI:** 10.3389/fbioe.2026.1923843

**Published:** 2026-10-19

**Authors:** Linus Weiß, Stefan Schneider, Kerstin Otte, Simon Fischer

**Affiliations:** 1 Institute of Applied Biotechnology, Biberach University of Applied Sciences, Biberach an der Riss, Germany; 2 Cell Line Development, Boehringer Ingelheim, Biberach an der Riss, Germany

**Keywords:** cell line engineering, Chinese hamster ovary cell, CRISPR Cas9 gene editing, hydrolase, multi-gene knockout, polysorbate degradation, transcriptomic profiling

## Abstract

CRISPR/Cas9-based multi-gene knockout strategies are widely applied to engineer Chinese hamster ovary (CHO) cells, yet the impact of editing workflow on cellular outcomes remains poorly understood. Here, we systematically compare sequential and multiplex genome editing approaches to generate CHO cell lines lacking nine polysorbate (PS)-degrading hydrolases. Both strategies achieved comparable functional outcomes, markedly improving PS stability. However, they produced distinct phenotypic and transcriptomic trajectories. Sequential editing led to progressive declines in growth and viability, increased cell size and DNA content, and extensive transcriptomic remodeling. In contrast, multiplex editing resulted in milder phenotypic alterations and a substantially smaller transcriptional footprint, albeit with increased clone-to-clone heterogeneity. Transcriptome analyses revealed limited overlap between final states, indicating workflow-specific adaptation despite the same intended knockout set. Importantly, transcriptional changes were primarily associated with clonal selection and iterative manipulation rather than reflecting a shared response to the targeted knockouts themselves. These findings demonstrate that genome editing workflows can shape cellular phenotypes independently of intended genetic modifications. For cell line development, multiplex editing offers a strategy to reduce development time and limit undesirable drift when targeting non-essential genes, highlighting the need to consider engineering strategy as a critical design parameter.

## 1 Introduction

The Chinese hamster ovary (CHO) cell remains the dominant mammalian host for the production of recombinant therapeutic proteins, accounting for the majority of approved monoclonal antibodies and many other biologics due to its favorable safety profile, robust growth in suspension culture, and capacity for human-compatible post-translational modifications (Kim et al., 2012; Walsh, 2018). Over the past four decades, cell line engineering has become a key strategy to further optimize CHO-based production platforms. Among the available engineering approaches, targeted gene knockout (KO) strategies have been particularly powerful for improving product quality, eliminating detrimental host cell proteins (HCPs), and enhancing cellular productivity (Fischer et al., 2015; Glinšek et al., 2023; Kalkan et al., 2023).

Early CHO cell engineering efforts primarily relied on random mutagenesis or RNA interference to suppress undesirable host cell functions (Fischer et al., 2015). The introduction of programmable genome editing technologies, enabled precise and efficient gene disruption and accelerated the use of KO-based engineering strategies in industrial cell line development (Ronda et al., 2014; Kalkan et al., 2023). Initial applications focused on single-gene KOs to remove specific HCPs or metabolic bottlenecks affecting biotherapeutic production. Representative examples include the disruption of alpha-(1,6)-fucosyltransferase 8 (*Fut8*) to generate afucosylated antibodies with enhanced antibody-dependent cellular cytotoxicity (Malphettes et al., 2010) and KOs of apoptosis regulators or metabolic enzymes to improve cell survival and productivity (Fischer et al., 2015; Glinšek et al., 2023).

More recently, CRISPR/Cas9 has made it possible to disrupt several genes within a single CHO cell line, either simultaneously or in sequence. Multi-gene KO strategies are particularly attractive when complex phenotypes depend on redundant gene families, when several problematic HCPs must be removed at once, or when compensatory mechanisms may limit the effect of a single-gene edit (Glinšek et al., 2023; Kalkan et al., 2023).

One area where multi-gene KO strategies have gained increasing attention is the mitigation of polysorbate (PS) degradation during the manufacturing of biologics. PS is a widely used surfactant in therapeutic protein formulations to prevent aggregation and surface adsorption (Wuchner et al., 2022; Felix et al., 2024). However, enzymatic degradation of PS can compromise product stability by generating free fatty acids that form visible or subvisible particles (Dwivedi et al., 2018). A growing body of evidence has demonstrated that PS degradation in CHO-based manufacturing processes is largely driven by host cell-derived hydrolases that retain activity during production and purification (Kovner et al., 2023; Felix et al., 2024; Maier et al., 2024). Several of these hydrolases have been identified as major contributors to PS hydrolysis. Consequently, targeted removal of PS-degrading enzymes has emerged as a promising strategy to stabilize PS during formulation and storage of therapeutic proteins (Chiu et al., 2017; Weiß et al., 2025; Yuk et al., 2025).

Multi-gene editing also introduces new challenges. Sequential editing workflows require repeated rounds of transfection, single-cell cloning, and prolonged cultivation, while multiplex editing introduces several genome perturbations at once and may require repeated transfections to achieve sufficient editing. Together with the inherent genomic plasticity of CHO cells, these steps can impose selective pressures and give rise to clones with altered growth, metabolism, or productivity (Wurm, 2013; Feichtinger et al., 2016; Weinguny et al., 2021). Genome editing itself can also introduce unintended genomic alterations, including off-target mutations or larger structural rearrangements after double-strand breaks (Kosicki et al., 2018). Systematic evaluation of editing workflows is therefore essential to distinguish effects caused by the intended KOs from those caused by the editing process itself.

In this study, we compared sequential editing and multiplex editing for the generation of CHO cell lines lacking multiple PS-degrading hydrolases. Because multiplex editing can reduce development time and repeated clone selection, we asked whether it also influences phenotypic and transcriptomic drift relative to sequential editing while achieving the same functional endpoint. By analyzing intermediate and final KO clones from both workflows, we examined KO-related effects and workflow-related adaptation using fed-batch phenotyping, targeted cellular assays, and RNA-seq.

## 2 Materials and methods

### 2.1 Cell culture

Boehringer Ingelheim proprietary CHO-K1 glutamine synthetase (GS)^−/−^ cell lines (female) adapted for suspension growth were cultivated in Boehringer proprietary chemically defined and animal component-free media. For standard cultivation, cells were cultured at 36.5 °C, 5% CO_2_, and 120 rpm (50 mm orbital throw) in an orbital shaker incubator (Infors) and passaged every 2–3 days.

### 2.2 Gene editing of CHO host cell line

Generation of sequential KO cell lines were performed as described previously (Weiß et al., 2025). For the multiplex approach, total sgRNA amount was kept constant, whereas dependent on the number of transfected sgRNAs amount of single sgRNAs was equally divided. Between multiplex transfections cells were grown for 3–7 days before re-transfection.

### 2.3 RNA sequencing

Gene expression analyses of fed-batch day 4 samples were performed by next-generation sequencing (NGS) as described previously (Weiß et al., 2025).

### 2.4 Fluorescence micelle assay

The assay was performed as described previously (Weiß et al., 2024). Cell culture supernatant was diluted in 0.4 mg/mL PS20 spiked, fresh cultivation media and incubated at room temperature. At different time points (d0, d1, d3, d7, d14), samples were frozen at −70 °C for simultaneous analysis.

### 2.5 Fed-batch cultivation

Fed-batch cultivations were performed using the ambr15® system as described previously (Weiß et al., 2025).

### 2.6 DNA index and cell cycle analysis

Cells were stained via Tali Cell Cycle Kit (Thermo Fisher) according to the manufacturer’s instruction and DNA content was analyzed via flow cytometry. Cell cycle phases were calculated via Watson Pragmatic model. The mean of G1 peaks were extracted and normalized to parental values to calculate the DNA index.

### 2.7 RNA-seq count processing and quality control

Gene-level count matrices were generated from aligned RNA-seq reads (as described previously (Weiß et al., 2025)) and imported into R (4.4.3) for downstream analysis. Samples were required to meet a minimum sequencing depth of 2.0 × 10^6^ assigned reads, libraries failing this threshold were excluded. All downstream statistical analyses were performed on the filtered set of retained samples, using gene identifiers that were consistently represented across the compared conditions.

### 2.8 Exploratory transcriptome analysis and low-dimensional embedding

To visualize global transcriptional relationships between parental and engineered CHO lineages, variance-stabilized expression values were computed using DESeq2 with standard size-factor estimation and variance-stabilizing transformation. UMAP was performed on the 5,000 most variable genes (by variance across samples) to emphasize biologically informative structure while reducing noise from low-variance features. UMAP was computed with the uwot implementation (min_dist = 0.2, fixed random seed) and the number of nearest neighbours set to min (15, n − 1) to ensure reproducibility.

### 2.9 Differential gene expression analysis

Differential expression (DE) testing was performed using a limma-voom framework with edgeR-based normalization. Raw counts were normalized using trimmed mean of M-values scaling to account for compositional differences between libraries. Genes with insufficient evidence of expression were filtered using *filterByExpr*, parameterized by the design matrix to retain features informative for the contrasts tested.

Normalized counts were transformed using *voomWithQualityWeights*, which estimates the mean-variance relationship and incorporates sample-specific quality weights. Linear models were fit using *lmFit*. Where clone-structured replication was present, within-clone correlation was modeled using *duplicateCorrelation*, and the resulting consensus correlation was propagated into the final model fit, thereby controlling for non-independence among samples derived from the same clone background. Contrasts were defined *a priori* to capture (i) stepwise changes across sequential KOs relative to parental, (ii) sequential/multiplex engineering relative to its parental reference, and (iii) direct comparisons between sequential and multiplex approaches. Moderated t-statistics were computed using empirical Bayes shrinkage. Multiple-testing correction was performed using the Benjamini–Hochberg (BH) procedure. DE gene sets were defined using a false discovery rate (FDR) < 0.05 and an effect-size cutoff of |log_2_ fold change| > 0.807 (corresponding to ∼1.75-fold).

### 2.10 Stepwise inheritance of differential expression across engineering stages

To quantify propagation of transcriptional changes across intermediate stages of sequential (S) and multiplex (M) engineering approaches, DE was computed at each stage relative to its corresponding parental reference using the *limma-voom* pipeline described above. Genes meeting FDR <0.05 and |log_2_ fold change| > 0.807 at a given stage were classified as newly differential if not previously detected as DE in earlier stages of the same engineering path, or as inherited if significant DE had been observed at any preceding stage in the same direction. Counts of newly differential versus inherited genes were summarized per stage and stratified by direction (up/down) to visualize accumulation and persistence of transcriptional changes across the engineering trajectories.

### 2.11 Functional enrichment analysis

Functional interpretation of DE gene sets was performed by over-representation analysis using *clusterProfiler*. DE gene lists were generated from the final-versus-parental contrasts, filtered at FDR <0.05, and |log_2_ fold change| > 0.807 (∼1.75-fold). Enrichment analysis was performed against a background universe comprising all genes retained after expression filtering in the corresponding limma-voom model described in Section 2.9. For analysis using human Gene Ontology (GO) resources, both the DE gene lists and their corresponding background universes were mapped from CHO gene identifiers to human NCBI Gene IDs using a curated orthology table. Enrichment testing was restricted to successfully mapped genes. P values were adjusted for multiple testing using the Benjamini–Hochberg procedure, and a minimum gene-set size of five was applied.

### 2.12 Shared differential expression analysis between final engineering endpoints

To identify a shared transcriptional core between final sequential and multiplex engineering endpoints, differential expression results from the final S-KO versus parental and final M-KO versus parental contrasts were filtered using the thresholds described above. Genes were retained when they were significantly differentially expressed in both workflows with FDR <0.05 and |log_2_ fold change| > 0.807 and showed concordant direction of regulation. For each shared gene, log_2_ fold changes from the final S-KO and final M-KO contrasts were extracted and compared directly. To visualize effect-size differences between workflows, final M-KO log_2_ fold changes were plotted against final S-KO log_2_ fold changes, with the diagonal indicating identical effect sizes between both endpoints. A conservative joint adjusted P value was calculated as the larger adjusted P value from the two contrasts. The complete data set underlying this comparison is provided in Supplementary Table S1.

### 2.13 Expression profiling of potential polysorbate-degrading hydrolases

To systematically assess potential transcriptional compensation following multi-hydrolase knockout, we examined expression changes across a curated panel of candidate polysorbate-degrading serine hydrolases. Candidate enzymes were operationally defined by annotated Ser, His, and Asp/Glu catalytic residues corresponding to the Ser-His-Asp/Glu catalytic triad previously associated with PS-active CHO-derived hydrolases (Maier et al., 2024). To curate a reference set of potential polysorbate-degrading hydrolases, human UniProtKB records were downloaded with Human GeneID (NCBI), protein name, gene name, active-site annotations and protein sequence. Active-site positions were parsed to extract annotated residue indices (including isoform-specific and range formats), residue identities were verified against the corresponding sequences, and ordered S-E-H or S-D-H triads (Ser < Asp/Glu < His) were called. The resulting triad-positive gene set was mapped to CHO genes via orthology and intersected with limma-voom differential expression results (FDR <0.05). The complete candidate panel of polysorbate-degrading serine hydrolases is provided in Supplementary Table S2.

## 3 Results

### 3.1 Generation of sequential and multiplex multi-KO cell lines

Single gene KOs in CHO cell line engineering have long been used to improve recombinant protein production and product quality (Fischer et al., 2015). For higher-order KOs, however, the same target gene set can be removed by different workflows. Sequential editing and multiplex editing workflows differ in timeline, number of electroporations, and number of clonal selection steps, which may influence the final cell state even when the intended KOs are identical.

We therefore compared both workflows by generating multi-hydrolase KO CHO cell lines designed to reduce PS degradation (Figure 1A). Sequential editing (S) started with the KO of *Lpl* and was followed by *Pla2g7*, combined *Pla2g15*/*Lipa*, Ces cluster 2, Ces cluster 1, and *Iah1*/*Ppt1*. Multiplex editing (M) started with the identical *Lpl* KO as in sequential editing, followed by stepwise removal of Ces cluster 2 and Ces cluster 1, after which *Pla2g7*, *Pla2g15*, *Lipa*, *Iah1*, and *Ppt1* were targeted together in repeated multiplex transfections. Individual gene KOs were introduced by Cas9-mediated double-strand break that generated insertion and/or deletion (indel) mutations predicted to cause loss of function, whereas the Ces clusters were removed by paired sgRNAs deleting genomic regions of 506 kb (Ces cluster 2) and 1,629 kb (Ces cluster 1). A stage-by-stage mapping of clone labels to newly introduced targets and cumulative KO sets is provided in Supplementary Table S3. Deleted genes within the excised Ces cluster 1 and 2 are listed in Supplementary Table S4.

**FIGURE 1:**
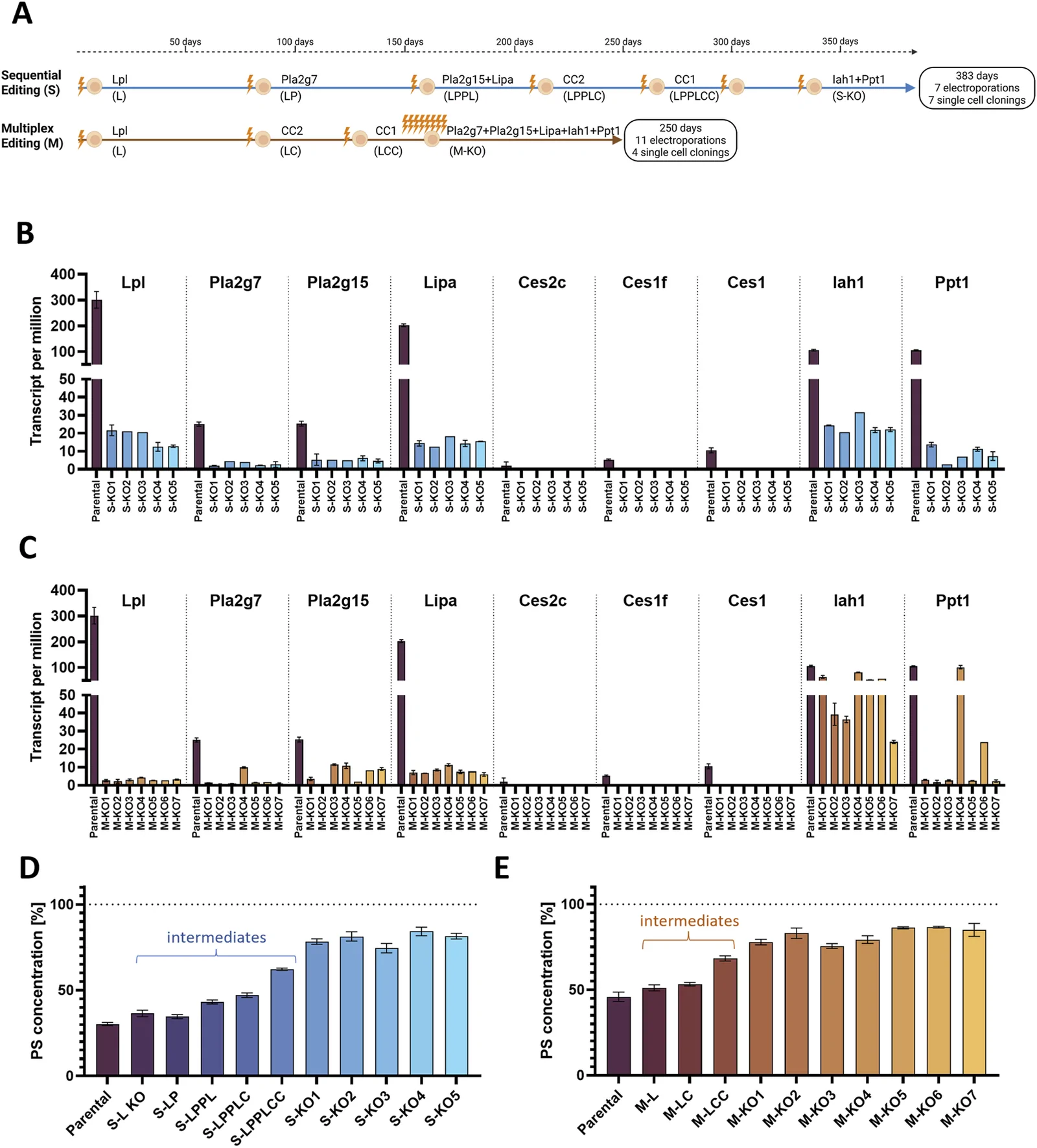
Sequential and multiplex workflows generate multi-hydrolase knockout (KO) CHO cells with convergent polysorbate (PS) stabilization. **(A)** Overview of the sequential (S) and multiplex (M) engineering strategies used to remove the same set of PS-degrading hydrolases. The sequential lineage introduced KOs stepwise, beginning with *Lpl*, followed by *Pla2g7*, combined *Pla2g15*/*Lipa*, Ces cluster 2, Ces cluster 1, and *Iah1*/*Ppt1*, resulting in final S-KO clones after 383 days, seven electroporations and seven single-cell cloning rounds. The multiplex lineage used the same initial *Lpl* KO and stepwise removal of both Ces clusters, followed by multiplex targeting of *Pla2g7*, *Pla2g15*, *Lipa*, *Iah1* and *Ppt1*, resulting in final M-KO clones after 250 days, eleven electroporations and four single-cell cloning rounds. **(B,C)** RNA-seq-based transcript abundance of the targeted hydrolases in parental and final sequential clones **(B)** or final multiplex clones **(C)**, shown as transcripts per million. Biological duplicates were measured (n = two independent fed-batch cultivations, Day 4); data are mean ± standard deviation. S-KO2, S-KO3 and M-KO6 were measured once (one representative fed-batch cultivation, Day 4) **(D,E)** Endpoint PS concentration in the fluorescence micelle assay across sequential intermediates and final S-KO clones **(D)** or multiplex intermediates and final M-KO clones **(E)**. Following pooling of biological duplicates, PS degradation over time was measured in four technical replicates. Values were normalized to initial PS concentration and are depicted as mean ± standard deviation.

The full sequential editing lineage has been described previously (Weiß et al., 2025) and required 383 days of cultivation, seven rounds of single cell cloning and seven electroporations to obtain final multi-KO clones (Figure 1A). Multiplex editing was started only after removal of both Ces clusters because these large genomic excisions were markedly less efficient. In total, multiplex editing needed a shorter time period of 250 days of cultivation, only four rounds of single cell cloning, but eleven electroporations to obtain final multi-KO clones (Figure 1A). The higher number of electroporations was caused by the usage of identical sgRNAs for both editing strategies, where low sgRNA efficiencies (Supplementary Figure S1A) posed a challenge for multiplex editing and required additional Cas9:sgRNA transfections (Supplementary Figure S1B). Here, the number of Cas9:sgRNA transfections was based on the editing rates of the respective target genes in the edited multiplex pools (Supplementary Figure S1C).

Editing outcomes in final sequential (S-KO1 to S-KO5) and multiplex (M-KO1 to M-KO7) clones were first assessed by RNA-seq. As expected, transcripts from the deleted Ces cluster genes were absent (Figures 1B,C). Most other loci targeted with single sgRNAs also showed strong transcript depletion, consistent with nonsense-mediated decay. Despite residual transcript signals detected in some multiplex clones for *Iah1* and *Ppt1*, successful editing at all intended loci was confirmed by Inference of CRISPR Edits (ICE) analysis in combination with PCR and gel electrophoresis (Supplementary Figures S2A–C).

Functional validation with a fluorescence-based micelle assay (Weiß et al., 2024) showed stepwise mitigation of PS degradation as additional hydrolases were removed (Figures 1D,E). PS retention increased from about 30% to 45% in the respective parental controls to more than 75% in final S-KO and M-KO clones.

Thus, both workflows generated CHO cell lines lacking the same nine PS-degrading hydrolases while exhibiting strongly improved PS retention. At the same time, the workflows differed in development time, number of electroporations and number of single-cell cloning rounds. In multiplex editing, all of these parameters, except the number of electroporations, were substantially reduced. This setup allowed a direct comparison of sequential and multiplex editing and allowed to test whether the editing workflow itself shapes the resulting phenotype and transcriptome.

### 3.2 Phenotypic impact of sequential and multiplex editing

To test the impact of sequential and multiplex hydrolase editing on bioprocess phenotypes, we compared intermediate and final hydrolase KO clones from both editing workflows in ambr15 fed-batch culture. In the sequential editing lineage, both peak viable cell density (VCD) and harvest viability declined in a stepwise manner as additional KOs were introduced (Figures 2A,B). Parental cultures reached peak VCDs of 25 × 10^6^ cells/mL, whereas final S-KO clones decreased to a range between 15–17 × 10^6^ cells/mL peak VCD. Harvest viabilities dropped from >90% in the parental line to 50%–60% in the final clones, except for S-KO2 with only 21%. In the multiplex lineage, growth also declined stepwise with increasing KO number, however, final clones retained better growth characteristics while showing more heterogeneous phenotypes after the multiplex KO step including seven electroporations (Figures 2C,D). These final M-KO clones ranged between 17 and 21 × 10^6^ cells/mL peak VCD, except for one clone specific alteration in M-KO4 with only 11 × 10^6^ cells/mL. Also harvest viabilities varied more but stayed higher than in final sequential clones with viabilities between 80% and 55%. Comparing both methods, sequential and multiplex workflows both led to a progressive decline in peak VCD and harvest viability with increasing KO number. However, these effects were consistently more pronounced and uniform in the sequential lineage, where final clones deviated stronger from the parental cell line. In contrast, multiplex editing resulted in overall milder average impacts on growth and viability, albeit with increased phenotypic heterogeneity among final clones following seven rounds of electroporation.

**FIGURE 2:**
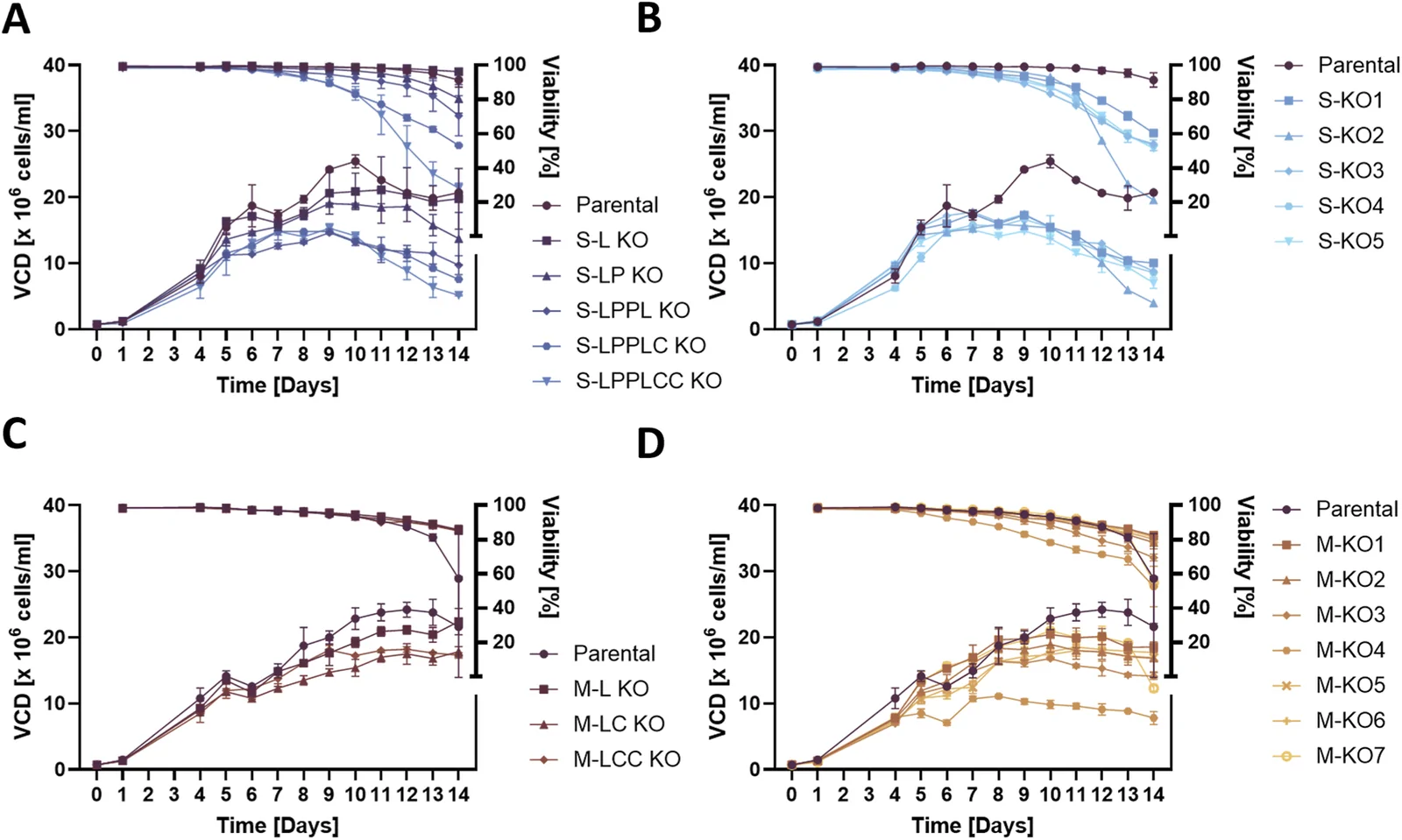
Sequential and multiplex hydrolase knockout (KO) workflows differ in their impact on fed-batch growth and viability. **(A–D)** Viable cell density (VCD) and viability profiles during 14 days fed-batch cultivation of sequential (S) intermediate clones **(A)**, final sequential S-KO clones **(B)**, multiplex (M) intermediate clones **(C)** and final multiplex M-KO clones **(D)**, with the parental CHO line included as a reference in each panel. All clones but S-KO2 and S-KO3 were cultivated in biological duplicates (n = 2); data are mean ± standard deviation.

For metabolic analysis, we first tested lactate levels which peaked on day 4 in all sequentially edited cultures (Figures 3A,B). After this peak, lactate levels decreased in all clones with the exception of the intermediate clones S-LP and S-LPPLC which showed compromised lactate clearance. Comparable to the sequential clones, lactate levels also peaked on day 4 in the multiplex lineage and homogeneously decreased in M-L and M-LC KO clones. After removal of Ces cluster 1 (M-LC to M-LCC) lactate levels remained elevated also in the final M-KO clones in comparison to parental cultures, indicating impaired lactate clearance (Figures 3C,D). Of note, other than for cell growth parameters, no stepwise shift in lactate metabolism was introduced per editing step and rather occurred randomly. Whereas alterations in lactate metabolism were partially reversible in the sequential workflow they were not reversible during later editing steps in the multiplex workflow.

**FIGURE 3:**
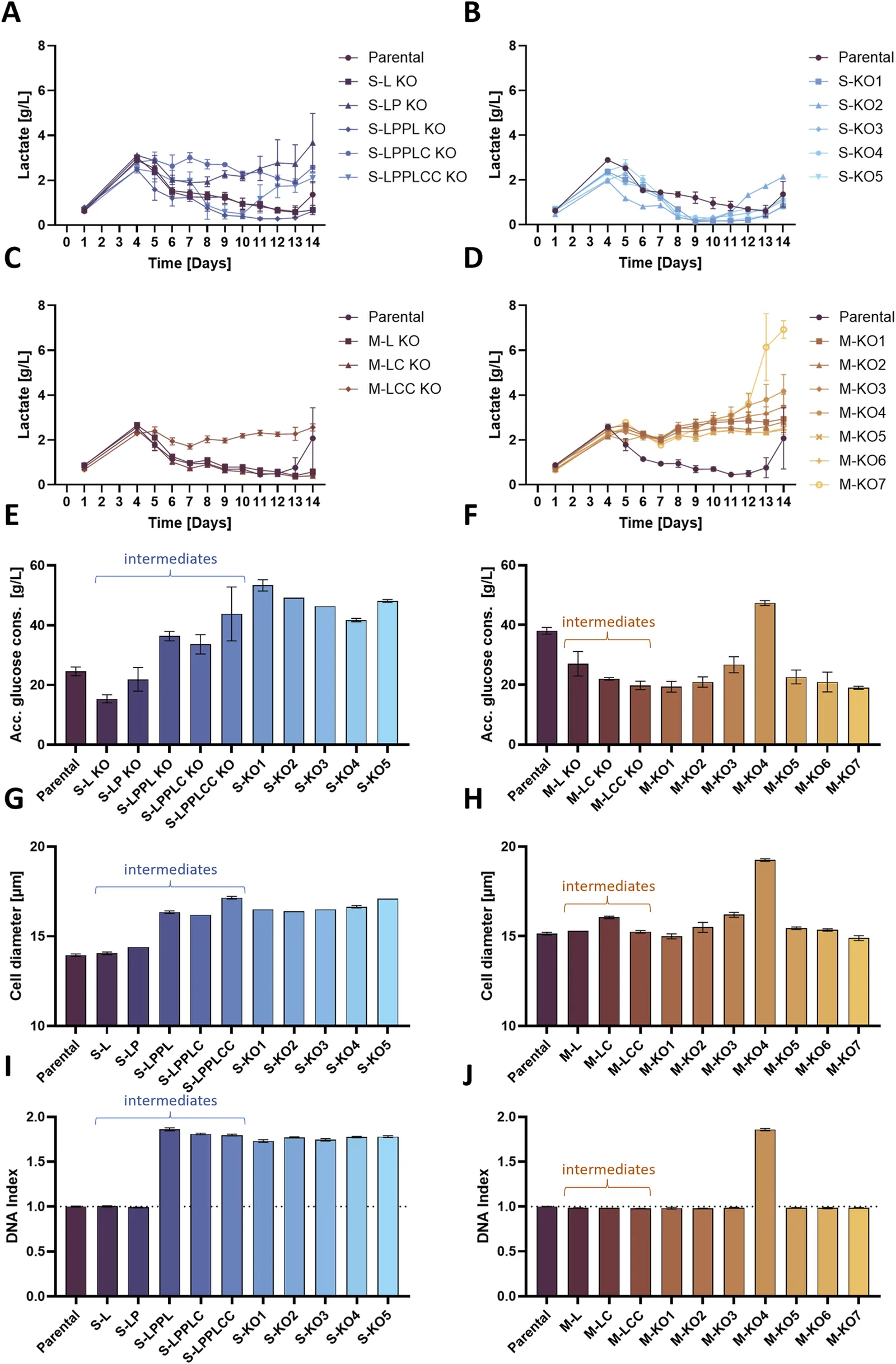
Metabolic and cellular physiology changes distinguish sequential and multiplex KO trajectories. **(A–D)** Lactate concentration profiles during 14 days fed-batch cultivation of sequential (S) intermediates **(A)**, final S-KO clones **(B)**, multiplex (M) intermediates **(C)** and final M-KO clones **(D)**. **(E,F)** Accumulated (Acc.) glucose consumption (cons.) at day 10 of fed-batch cultivations of sequential **(E)** and multiplex **(F)** lineages. **(G,H)** Cell diameter of sequential **(G)** and multiplex **(H)** lineages. **(I,J)** DNA index normalized to the parental control in sequential **(I)** and multiplex **(J)** lineages. Dotted horizontal lines indicate the parental DNA index. All clones but S-KO2 and S-KO3 were cultivated in biological duplicates (n = 2); data are mean ± standard deviation. DNA index was measured in technical triplicates.

Accumulated (Acc.) glucose consumption (cons.) on day 10 (end of exponential growth phase) decreased in the first sequential editing step (Parental → S-L KO) from 25 g/L to 15 g/L followed by large, stepwise increases towards the final S-KO clones, which varied between 53 g/L and 42 g/L (Figure 3E). Similarly, the initial editing step in the multiplex workflow (Parental → M-L KO) caused a decrease in acc. glucose cons. from 38 g/L to 27 g/L. In contrast to the sequential workflow, acc. glucose cons. decreased slightly towards the final M-KO clones, which varied between 19 g/L and 27 g/L, except for one clone-specific alteration in clone M-KO4 with 47 g/L (Figure 3F). Metabolic analyses revealed that changes in lactate handling and glucose consumption did not follow a strictly stepwise pattern and appeared decoupled from KO number and target gene, suggesting clone-specific rather than cumulative pathway disruption.

When analyzing the diameter of edited cell lines, KO of *Pla2g15* and *Lipa* (S-LP → S-LPPL) in the sequential editing lineage coincided with an increase in cell diameter relative to the parental CHO cell line (Figure 3G). This shift was not reversed through the editing workflow. In the multiplex lineage, this increase in cell diameter was not observed in corresponding multiplex edited clones, however, M-KO4 again was the exception displaying a clone-specific event of diameter increase (Figure 3H). Interestingly, cell diameter correlated with increased DNA content for the sequential lineage beginning with S-LP to S-LPPL KO and the multiplex clone M-KO4 (Figures 3I,J). Moreover, increased cell diameter and DNA content correlated with reduced proliferation, which was observed in the S-LPPL KO clone extending to higher-order sequential clones and clone M-KO4 (Figures 2A–D). The observed shifts in cell diameter and DNA content in sequentially edited clones and in clone M-KO4 further support the notion that repeated electroporation, cloning, and expansion steps harbor the risk of clone-specific, rather than KO target-specific, disruptions, leading to unfavorable bioprocessing traits.

Together, these results demonstrate that extensive hydrolase KOs induce measurable trade-offs in growth, metabolism, and cell physiology, with the editing strategy itself exerting a significant influence on phenotypic severity. The stepwise and partly random accumulation of unfavorable phenotypic traits supports clone selection and repeated single-cell isolation as major contributors to these changes. While repeated electroporation may add heterogeneity, the number of electroporation events alone does not explain the pattern as it was higher in the multiplex workflow.

### 3.3 Transcriptome-wide consequences of sequential versus multiplex editing

To quantify the global impact on the transcriptome of the two editing workflows beyond the intended hydrolase disruptions and phenotypic data, all above-described cell clones were profiled by RNA-seq and transcriptional states compared within and across lineages. In the final S-KO clones, differential expression relative to the corresponding parental control was extensive, with 425 upregulated and 415 downregulated genes (Figure 4A). In contrast, final M-KO clones exhibited a markedly smaller response (162 upregulated and 109 downregulated genes; Figure 4B). Direct comparison of the final S-KO and M-KO clones further showed broad transcriptional separation between the two endpoints, with 894 genes higher and 1,003 genes lower in the final S-KO clones versus the final M-KO clones (Figure 4C). Thus, despite engineering toward a shared functional objective, the sequential workflow produced a substantially larger, and largely different transcriptional footprint than the multiplex workflow. This observation aligns with a larger decrease of average cell growth parameters (VCD and viability) in the sequential workflow than in the multiplex workflow.

**FIGURE 4:**
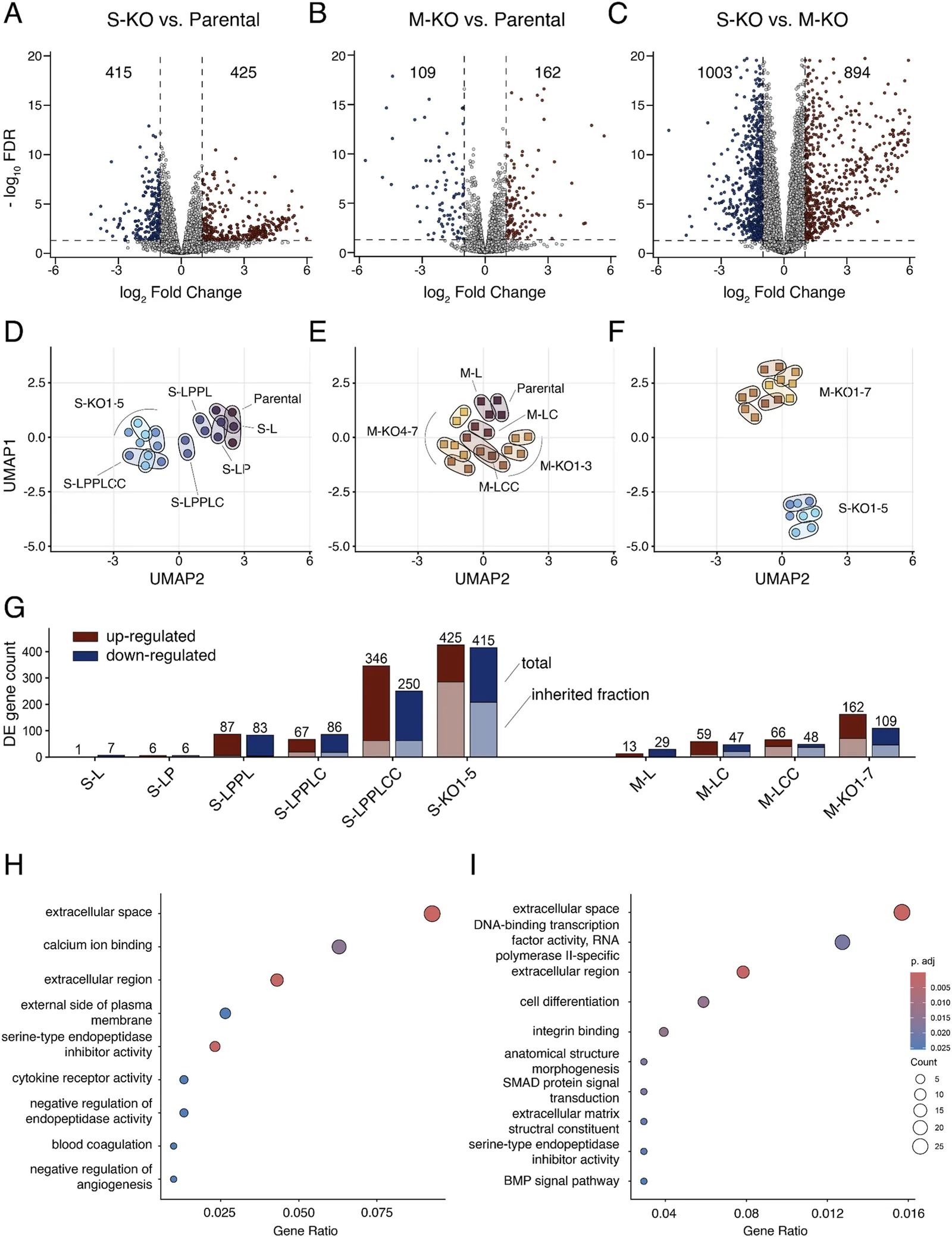
Sequential editing produces a larger and distinct transcriptomic footprint than multiplex editing. **(A–C)** Volcano plots showing differential gene expression in final Sequential (S)-KO clones versus parental controls **(A)**, final Multiplex (M)-KO clones versus parental controls **(B)** and final S-KO versus final M-KO clones **(C)**. Dashed lines mark the false-discovery-rate (FDR <0.05) and effect-size (|log_2_FC| > 0.807, ≈1.75-fold) thresholds defining differential expression; red and blue denote up- and downregulated genes meeting both thresholds. **(D–F)** Uniform Manifold Approximation and Projection (UMAP) embeddings of variance-stabilized RNA-seq profiles for the sequential lineage **(D)**, multiplex lineage **(E)** and final S-KO and M-KO clones analyzed together **(F)**. Point fill denotes editing step and point shape denotes editing approach (circle, sequential; rectangle multiplex). Each point represents one RNA-seq library; enveloped points mark the two cultivation replicates of a clone that was measured in biological duplicate (n = two independent fed-batch cultivations, Day 4), whereas non-enveloped points represent clones measured once (S-KO2, S-KO3 and M-KO6). **(G)** Stage-resolved differential expression (DE) across the sequential and multiplex engineering trajectories. Full bar height indicates the total number of upregulated or downregulated DE genes at each stage, and lighter segments indicate the fraction inherited from previous stages. **(H,I)** Gene ontology (GO) over-representation analysis of upregulated genes (FDR <0.05, log_2_FC > 0.807) in final S-KO clones **(H)** and final M-KO clones **(I)**. Dot size denotes gene count, and color denotes adjusted P value (p.adj). Final S-KO comprises 5 clones (S-KO1-5; 8 libraries) and final M-KO comprises 7 clones (M-KO1-7; 13 libraries); each intermediate stage and parental control is a single clone profiled in two fed-batch cultivation replicates except S-KO2, S-KO3 and M-KO6, which were profiled once.

To visualize transcriptomic variability between samples and approaches, each RNA-seq profile was projected onto a two-dimensional uniform manifold approximation and projection (UMAP) map, where nearby points indicate more similar transcriptomes. Along the sequential lineage, intermediate clones moved stepwise away from the parental state toward the final S-KO cluster, consistent with progressive transcriptomic and phenotypic drift across repeated electroporation, cloning, and expansion steps (Figure 4D). In contrast, the multiplex lineage occupied a more confined transcriptional space throughout the engineering process (Figure 4E), in accordance with reduced phenotypic deviation from the parental cell line, in particular concerning growth and viability. When both lineages were analyzed together, final S-KO and M-KO clones formed separate clusters (Figure 4F), indicating that the two workflows reached different transcriptomic endpoints despite the same intended KO set.

We next asked whether the larger transcriptional divergence observed in the sequential workflow reflects the cumulative burden of genome editing and associated genome remodeling *per se*, or instead is dominated by clone selection during repeated single-cell isolation. Therefore, it was quantified how many differentially expressed (DE) genes newly appeared at each stage and persisted (‘inherited’) from earlier stages. Stage-resolved DE counts with inheritance tracking showed that the earliest sequential intermediates changed only modestly (S-L: 1 up/7 down; S-LP: 6 up/6 down), whereas introduction of the *Pla2g15* and *Lipa* (S-LP → S-LPPL) KOs marked the first pronounced increase in differentially expressed genes (87 up/83 down; Figure 4G). Notably, this transcriptional step-up coincided with the inherited increase in DNA content and cell size described above. The following Ces cluster 2 deletion (S-LPPL → S-LPPLC) did not substantially increase the overall response further (67 up/86 down). A much larger difference appeared after deletion of Ces cluster one at S-LPPLCC (346 up/250 down). Analysis of genes flanking either Ces-cluster deletion did not reveal broad local expression changes that could account for the genome-wide increase in DE genes, arguing against deletion-associated position effects as the principal driver (Supplementary Figure S3). This transcriptomic step-up was not matched by a comparably broad new phenotypic transition, as observed at S-LPPL. Many of these changes persisted in final S-KO clones (425 up/415 down). In the multiplex lineage, transcriptional changes remained smaller throughout, with 13 up/29 down in M-L, 59 up/47 down in M-LC, 66 up/48 down in M-LCC, and 162 up/109 down in the final M-KO clones (Figure 4G). At the lineage level, this smaller and more gradual transcriptome shift matched the milder phenotype changes seen in fed-batch culture. Notably, the largest transcriptional changes were observed in the final multiplex KO step (M-LCC → M-KO1-7) including seven electroporation events. Nevertheless, the magnitude of this increase was not greater than some of the changes observed in the sequential workflow. Together, these patterns argue that the large shift observed in the sequential lineage is unlikely to be explained by the Ces-cluster 1 deletion event alone. Instead, it points to clonal adaption, clone selection and repeated single-cell isolation as a major driver of the final transcriptional state, whereas the number of electroporation events seem to play only a secondary role.

Over-representation analysis of the upregulated gene sets further emphasized that the two workflows elicited different biological responses. In final S-KO clones, enriched GO terms were dominated by extracellular and secreted-factor related annotations, including extracellular space, extracellular region, external side of plasma membrane, serine-type endopeptidase inhibitor activity, and cytokine receptor activity (Figure 4H). Final M-KO clones also showed extracellular enrichment, but additionally displayed terms linked to transcriptional regulation and developmental signaling, including DNA-binding transcription factor activity, cell differentiation, integrin binding, SMAD protein signal transduction, and BMP signal pathway (Figure 4I). Thus, both workflows shared an extracellular component, whereas the broader regulatory signature was more prominent in the multiplex endpoint. Because these overlapping extracellular terms could either reflect a true shared core gene set or distinct gene sets reaching similar annotations, we next examined the concordantly regulated genes shared between both final states.

### 3.4 Divergent transcriptional adaptation despite convergent polysorbate stabilization

Given the clear separation of the final transcriptomic states, we next asked whether both workflows retained a common transcriptional core that might reflect a shared response to broad hydrolase loss. Using a stringent cutoff (adjusted P < 0.05 and |log_2_FC| > 0.807), overlap between the final S-KO and M-KO states was limited. Among upregulated genes, only 50 were shared, whereas 375 were unique to the sequential workflow and 112 were unique to the multiplex workflow (Figure 5A). The same pattern was observed for downregulated genes, where only 15 genes overlapped, compared with 400 sequential-specific and 94 multiplex-specific genes (Figure 5A). Even within the shared set, effect sizes often deviated substantially from a 1:1 relationship between workflows (Figure 5B). Inspection of the concordantly upregulated overlap genes (Supplementary Table S1; Figure 5B) showed that this small, shared core was not functionally random. Several genes encode extracellular or cell-surface factors, including *Cd109*, *Serpine1*, *Fn1*, *Angptl4*, *Csf1*, and *F7*, consistent with the extracellular GO terms observed in both workflows (Figures 4H,I). A smaller shared regulatory subset, including *Prrx1*, *Notch1*, *Smad6*, and *Dtx4*, may account for the limited recurrence of signaling-related annotations. Together, these data suggest that broad hydrolase loss may trigger a limited common extracellular remodeling response, but that most of the final transcriptional adaptation remains strongly workflow-specific.

**FIGURE 5:**
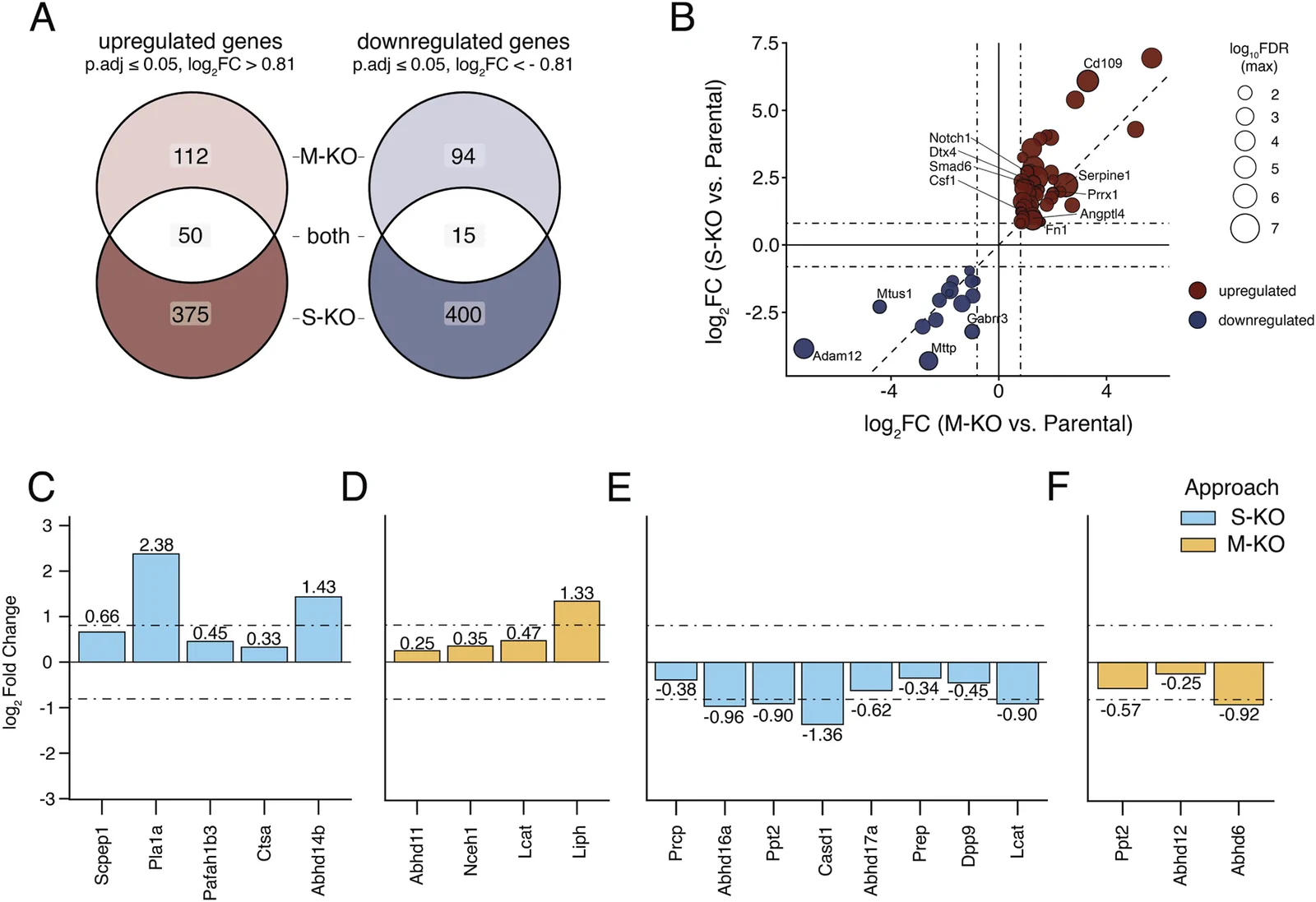
Final sequential and multiplex endpoints share a limited transcriptional core and lack broad hydrolase compensation. **(A)** Overlap of upregulated (red) and downregulated (blue) genes in final Sequential (S)-KO and Multiplex (M)-KO clones relative to parental controls (adjusted P ≤ 0.05 and |log_2_FC| > 0.807). **(B)** Effect-size comparison of genes differentially expressed in both final endpoints. Axes show log_2_FC relative to parental controls for M-KO and S-KO clones, and the diagonal indicates equal effect size between workflows. Point size denotes the maximum FDR-derived significance across the two contrasts, and selected shared extracellular, cell-surface and regulatory genes are labelled. **(C–F)** Expression changes of curated candidate Polysorbate-degrading serine hydrolases in final S-KO (blue) **(C,E)** and M-KO **(D,F)** clones (yellow). Bars show log_2_ Fold Change relative to parental controls for hydrolases with positive **(C,D)** or negative **(E,F)** expression shifts. Dashed horizontal lines indicate the |log_2_FC| = 0.807 threshold. Final S-KO comprises 5 clones (S-KO1-5; 8 libraries) and final M-KO comprises 7 clones (M-KO1-7; 13 libraries); and parental control is a single clone profiled in two fed-batch cultivation replicates. S-KO2, S-KO3 and M-KO6 were measured once.

A key concern in PS-stability engineering is that eliminating dominant PS-degrading hydrolases could induce compensatory upregulation of alternative hydrolases, thereby potentially diminishing the engineered effect. To systematically assess potential compensatory responses, we examined expression changes across a curated panel of candidate PS-degrading serine hydrolases, defined by the conserved Ser-His-Asp/Glu catalytic triad previously associated with active CHO-derived hydrolases (Supplementary Table S2; Figures 5C–F) (Maier et al., 2024). The complete curated panel is listed in Supplementary Table S2, whereas Figures 5C–F shows only genes from this panel that were significantly differentially expressed in the final S-KO or final M-KO clones (adjusted P ≤ 0.05). Across both workflows, most genes showed minimal deviation from baseline or shifts above the fold-change threshold (|log_2_FC| > 0.807), providing no evidence for broad transcriptional compensation (Figures 5C–F). In final S-KO clones, *Pla1a* (log_2_FC 2.38) and *Abhd14b* (1.43) were the only hydrolases clearly upregulated above threshold, whereas in final M-KO clones *Liph* (1.33) was the only such hit (Figures 5C,D). Most remaining hydrolases showed only modest positive changes. Several hydrolases decreased in the sequential lineage, including *Abhd16a*, *Ppt2*, *Casd1*, and *Lcat*, whereas the multiplex lineage showed fewer downward shifts, with Abhd6 representing the only candidate dropping below the fold-change threshold (|log_2_FC| > 0.807) in the final M-KO clones (Figures 5E,F).

Notably, the most consistent hydrolase-linked relationship across both workflows involved the palmitoyl-thioesterase family: *Ppt2*, a lysosomal thioesterase paralog of *Ppt1* with distinct substrate specificity, was reduced in both final states (Figures 5E,F). This pattern is opposite to what would be expected under a simple transcript-level compensation model in which loss of *Ppt1* is buffered by induction of a closely related thioesterase.

Overall, these data provide little evidence for a broad, shared transcriptional compensation program after multi-hydrolase KO. Rather, the final sequential and multiplex clones shared the intended hydrolase-KO genotype and converged on the desired PS-stabilizing phenotype but differed in the extent of workflow-associated drift. Sequential engineering produced the larger difference from the parental state, marked by impaired growth and viability, altered glucose consumption, increased cell diameter and a broader transcriptomic footprint. Multiplex engineering, in contrast, maintained a more confined transcriptional trajectory and generally milder phenotypic changes, despite clone-to-clone heterogeneity after the final multiplex editing step. For cell line development, this genotype-phenotype comparison favors multiplex KO for non-detrimental HCP target sets, as it achieves the same product-quality endpoint with fewer cloning rounds and shorter development time while limiting unwanted clonal drift.

## 4 Discussion

This study provides a systematic comparison of sequential and multiplex CRISPR/Cas9-based KO strategies in CHO cells that ultimately generate identical target gene KOs but differ substantially in their engineering workflows. While both approaches successfully produced cell lines lacking nine PS-degrading hydrolases and achieved comparable stabilization of PS, the resulting clones exhibited markedly different phenotypic and transcriptional trajectories during their generation. These findings demonstrate that the genome editing strategy itself can substantially influence cellular adaptation during cell line engineering and should therefore be considered alongside the intended genetic modifications.

Sequential KO clones displayed a considerably larger transcriptional footprint and progressive deterioration of growth characteristics, including reduced proliferation and decreased viability. Multiplex KO clones also showed workflow-associated alterations, most notably impaired lactate clearance in the final state and the clone-specific M-KO4 outlier, but maintained a smaller overall transcriptional deviation from the parental state and, with the exception of M-KO4, milder changes in growth, cell diameter and DNA index. These observations are consistent with the inherent genomic plasticity of CHO cells, which undergo continuous structural and transcriptional evolution during prolonged cultivation, manipulation steps and clonal selection (Lewis et al., 2013; Wurm, 2013; Feichtinger et al., 2016). Sequential genome editing requires multiple cycles of electroporation and single-cell cloning and clonal expansion, which imposes selective pressures that can drive genetic drift and transcriptional divergence. In agreement with this interpretation, stage-resolved transcriptomic analysis indicated that major transcriptional shifts were primarily associated with clonal selection steps rather than with individual editing events themselves, or prolonged cultivation duration. This is in agreement with Feichtinger and colleagues, who attributed genotypical and phenotypic drifts to perturbations during cultivation rather than prolonged time in culture (Feichtinger et al., 2016). The importance of clone selection is further supported by the more compact transcriptomic trajectory of the multiplex lineage, which experienced fewer cycles of single-cell cloning, although the M-KO4 phenotype shows that clone-specific alterations can still arise after multiplex editing. Previous studies have similarly shown that subclones derived from the same parental CHO population can diverge substantially in gene expression and phenotypic performance as a result of clonal selection alone (Pilbrough et al., 2009; Feichtinger et al., 2016; Chen et al., 2017; Morina et al., 2025).

A mock-editing workflow with non-targeting sgRNAs could further characterize the range of variation associated with repeated manipulation and single-cell cloning. Because each mock lineage would itself pass through independent clonal bottlenecks and selection, however, it would define a distribution of workflow-associated outcomes rather than a single fixed baseline from which clonal and editing effects could be directly subtracted. Such a control would therefore add useful context but would not, by itself, assign each observed change to one component. Nevertheless, the same intended KO set, limited overlap between final transcriptomic states, different stage-resolved trajectories and smaller multiplex footprint despite more electroporations support editing workflow and clonal history as major contributors to the divergent outcomes.

In the present workflow, re-use of sgRNAs that were sufficient for sequential editing resulted in low per-target efficiencies for several loci and necessitated repeated multiplex transfections. Thus, high-efficiency sgRNA panels are not merely beneficial but foundational for realizing the time-saving potential of multiplex KO. This observation is consistent with a previous multiplex editing study in CHO cells showing that the frequency of clones with the desired genotype decreases substantially as the number of targeted loci increases (Carver et al., 2022).

Another notable finding is that phenotypic as well as transcriptomic changes emerging during the engineering process were rarely reversible in subsequent editing steps. Sequential KO clones displayed progressive alterations including increased cell diameter, altered metabolic behavior, reduced proliferation rates and accumulating DE genes. Once established, these traits mostly persisted throughout the remaining engineering trajectory. Dhiman and colleagues explained differences in phenotypes by a mixture of mutations in relevant genes and changes in their expression patterns (Dhiman et al., 2019). Therefore, the observed changes in phenotypes and transcriptome probably reflect stable genetic, epigenetic or regulatory alterations acquired during each manipulation step, which are unlikely to be reversed unless they impose strong selective pressure. Similar phenotypic and genotypic drift during iterative perturbation steps has been reported previously and represents a recognized challenge in CHO cell line development (Feichtinger et al., 2016; Weinguny et al., 2021). These findings emphasize the importance of early comprehensive screening, careful clone selection and characterization as unfavorable traits introduced during early engineering stages may propagate through the entire lineage of derived clones.

Deletion of multiple PS-degrading hydrolases raises the possibility that compensatory mechanisms could restore PS degradation through upregulation of alternative enzymes. However, transcriptomic analysis provided limited evidence for a universal compensatory response. This is in line with the work of Weinguny and colleagues, who observed large transcriptional differences of individual clones subjected to identical selective pressure (Weinguny et al., 2020). They conclude that a cell has multiple ways to adapt given numerous genetic and subsequently metabolic redundancies. Only a small number of candidate hydrolases showed increased expression in the final KO clones, and these responses differed between the sequential and multiplex workflows. For example, *Abhd14b* and *Pla1a* were upregulated in sequential KO clones, whereas *Liph* was increased in multiplex clones. Because these responses were workflow-specific rather than shared between both strategies, they likely reflect lineage-specific transcriptional adaptations rather than general compensatory mechanisms. Interestingly, the lysosomal thioesterase paralog *Ppt2* was consistently downregulated in both final KO populations despite KO of its paralog *Ppt1*, which does not support a simple compensation model between these enzymes. Instead, the data suggest that multiple transcriptional trajectories can accommodate hydrolase loss while maintaining the desired PS-stabilizing phenotype. Consistent with this interpretation, none of the observed phenotypic or transcriptomic alterations could be unequivocally attributed to disruption of a specific hydrolase or hydrolase combination. Nevertheless, the workflow-specific upregulation of individual candidate hydrolases, including *Pla1a* and *Abhd14b* in S-KO clones and Liph in M-KO clones, underscores that targeted hydrolase-expression screening remains important during clone selection. This indicates that the engineering process itself represents the dominant driver of cellular adaptation and further supports that hydrolase KOs are a valid strategy to tackle PS degradation in biopharmaceutical production processes (Weiß et al., 2025; Yuk et al., 2025).

The comparison of sequential and multiplex editing strategies highlights distinct advantages and limitations for both approaches. Sequential editing offers a controlled and stepwise engineering path that may be advantageous when targeting genes with potential synthetic lethality or essential cellular functions, as intermediate phenotypes can be monitored before additional genes are disrupted. Multiplex editing, in contrast, substantially reduces development timelines, the number of clonal selection steps required to generate the desired genotype and displays a reduced phenotypic and genotypical drift compared with the sequential strategy. However, multiplex success depends strongly on sgRNA efficiency and genotype-first screening, because low-efficiency guides reduce the probability of isolating clones carrying complete biallelic disruption across all intended loci. Multiplex editing also does not eliminate clone-specific alterations, as illustrated by M-KO4, so final clone selection must still include phenotypic and PS-stability screening. The choice between sequential and multiplex strategies therefore represents a trade-off between engineering time, screening effort, and control over intermediate phenotypes. As multi-gene KO approaches will become increasingly common in cell line development, comprehensive genomic and multi-omics analyses are necessary to better understand the consequences associated with the combined KO of multiple genes in a single CHO cell line.

In summary, this study demonstrates that sequential and multiplex genome editing strategies can introduce the same hydrolase-KO genotype and achieve the same PS-stabilizing phenotype while producing distinct phenotypic and transcriptional trajectories in CHO cells. For target sets whose loss is expected to be compatible with host-cell fitness, such as non-essential HCPs, the data favor multiplex KO as the preferred development strategy because it reached the same functional endpoint with shorter development time and fewer single-cell cloning rounds, while limiting the accumulated growth, DNA-index and transcriptomic drift observed in the sequential lineage. Sequential editing remains valuable when target biology is uncertain or when removal of individual genes may impair essential cellular functions, because intermediate phenotypes can be assessed before additional KOs are introduced. Irrespective of the editing workflow, final clone selection should combine genotype confirmation, fed-batch performance assessment and PS-stability screening to enable robust cell line performance.

## Data Availability

The datasets presented in this article are not readily available because the underlying RNA-seq data contain proprietary and confidential information of Boehringer Ingelheim and are subject to legal restrictions on disclosure. Requests to access the datasets should be directed to the corresponding author.
