# Supplementary material for: Same hydrolase knockouts, different outcomes: CRISPR/Cas9 editing workflow shapes resulting cellular phenotype and transcriptome in CHO
Source: Front Bioeng Biotechnol. 2026 Oct 19;14:1923843. doi: 10.3389/fbioe.2026.1923843 (PMC13724508; doi:10.3389/fbioe.2026.1923843)
Supplement: Supplementary file 1 [file Supplementaryfile1.docx]

**Supplementary Table 1: Concordantly regulated overlap genes shared between final sequential and multiplex knockout endpoints**

Genes significantly differentially expressed in both final S-KO and final M-KO clones relative to their parental controls and showing the same direction of regulation. The table includes CHO GeneID (NCBI), gene symbol, direction, workflow-specific log₂ fold changes, adjusted P values, joint significance scores, mean log₂ fold change and S-KO-M-KO effect-size differences.

**Supplementary Table 2: Curated panel of candidate polysorbate-degrading serine hydrolases**

Curated reference panel of candidate polysorbate-degrading serine hydrolases used to assess transcriptional compensation after multi-hydrolase knockout. Candidate enzymes were defined by annotated catalytic Ser, His and Asp/Glu residues corresponding to an ordered S-D-H or S-E-H catalytic triad, as described in the Methods (Section 2.14). The table includes human GeneID (NCBI), protein name, annotated active-site residues, human gene name, mapped CHO gene name and CHO GeneID (NCBI).
