# Supplementary material for: Same hydrolase knockouts, different outcomes: CRISPR/Cas9 editing workflow shapes resulting cellular phenotype and transcriptome in CHO
Source: Front Bioeng Biotechnol. 2026 Oct 19;14:1923843. doi: 10.3389/fbioe.2026.1923843 (PMC13724508; doi:10.3389/fbioe.2026.1923843)
Supplement: Supplementary file 2 [file Table3.docx]

Supplementary Tables

**Supplementary Table 3**: Clone-stage labels with knockout set for sequential and multiplex engineering. Labels are mapped to the newly introduced target or targets at each stage and to the cumulative intended knockout set.

| **Workflow** | **Stage label** | **Newly introduced target(s)** | **Knockout set** |
| --- | --- | --- | --- |
| Sequential | S-L | Lpl | Lpl |
| Sequential | S-LP | Pla2g7 | Lpl; Pla2g7 |
| Sequential | S-LPPL | Pla2g15; Lipa | Lpl; Pla2g7; Pla2g15; Lipa |
| Sequential | S-LPPLC | Ces cluster 2 (Ces2c) | Lpl; Pla2g7; Pla2g15; Lipa; Ces2c |
| Sequential | S-LPPLCC | Ces cluster 1 (Ces1; Ces1f) | Lpl; Pla2g7; Pla2g15; Lipa; Ces2c; Ces1; Ces1f |
| Sequential | S-KO1–S-KO5 | Iah1; Ppt1 | Lpl; Pla2g7; Pla2g15; Lipa; Ces2c; Ces1; Ces1f; Iah1; Ppt1 |
| Multiplex | M-L | Lpl | Lpl |
| Multiplex | M-LC | Ces cluster 2 (Ces2c) | Lpl; Ces2c |
| Multiplex | M-LCC | Ces cluster 1 (Ces1; Ces1f) | Lpl; Ces2c; Ces1; Ces1f |
| Multiplex | M-KO1–M-KO7 | Pla2g7; Pla2g15; Lipa; Iah1; Ppt1 | Lpl; Ces2c; Ces1; Ces1f; Pla2g7; Pla2g15; Lipa; Iah1; Ppt1 |

**Supplementary Table 4**: Deleted genes within the excised Ces-cluster 1 and 2. For reader convenience, the gene labels are reproduced in left-to-right order from the detailed structural schematics in our previous characterization of these intervals (Weiß et al., 2025; doi:10.1016/j.tibtech.2025.04.016, Figure S1). Labels follow Weiß et al. (2025), Figure S1; V denotes the serial variant designation used in that source.

| **Deleted region** | **Deleted genes** |
| --- | --- |
| Ces cluster 1 | Ces5a; Ces1d; Ces1e V1; Ces1f V1; Ces1 V1; Ces1 V2; Ces1 V3; Ces1f V2; Ces1e V2; Ces V1; Ces V2; Ces1e V3; Ces1c; Ces V3; Slc6a2; Lpcat2 V1; Capns2; Lpcat2 V2; Mmp2; Irx6 |
| Ces cluster 2 | Ces2c V1; Ces V1; Ces V2; Ces V3; Ces V4; Ces V5; Ces V6; Env; Smad1; Ces2c V2; Ces2c V3; Ces2e V1; Ces V7; Ces2c V4; Ces2c V5; Ces2e V2; Rpl7; Ces2a; Amd1; Ces3b; Ces4a |
